# Optimization and Evaluation of the *Quercus infectoria* Galls Thermosensitive In Situ Gel for Rectal Delivery

**DOI:** 10.1155/2022/8451055

**Published:** 2022-10-03

**Authors:** Abdulaziz Arkin, Aliya Elham, Arfidin Anwar, Gulina Kalimanjiang, Mubarak Iminjan

**Affiliations:** Department of Pharmaceutics and Physical Chemistry, College of Pharmacy, Xinjiang Medical University, Urumqi 830017, China

## Abstract

*Quercus infectoria* galls (QIGs) have a long history of treating ulcerative colitis (UC). The aqueous extract of QIG has an anti-UC effect. However, QIG's enema is easy to leak, and the action time and dose of the drug cannot be controlled well. Thus, QIG is inconvenient to use. This study aims to screen and prepare an optimized thermosensitive in situ gel with slow release and retention. Taking the transition sol-gel temperature (*T*_sol-gel_) as the investigation index, the Box-Behnken design response surface method (BBD-RSM) was used to optimize the dosages of Poloxamer 407 (P407), Poloxamer 188 (P188), and hydroxypropyl methyl cellulose (HPMC). Moreover, three formulations were selected, and the *in vitro* release rates were further optimized. The optimized rates of P407, P188, and HPMC were 24.07%, 1.22%, and 0.60%, respectively, and *T*_sol-gel_ was 32.8°C ± 0.4°C. The cumulative release of gallic acid in the gel conformed to the first-order kinetic equation, and gallic acid was released entirely within 24 h. In addition, the morphological and chemical characterization of thermosensitive in situ gel demonstrated that excipients did not affect the characteristic functional groups of QIG and that the surface of the QIG gel had a porous and loose structure. Rheological methods showed that the QIG thermosensitive in situ gel was fluid at low temperature and semisolid at gelation temperature. Therefore, the prepared gel was sensitive to temperature and had slow-release, local retention properties.

## 1. Introduction

Ulcerative colitis (UC), a nonspecific disease occurring in the colorectal mucosa, is a type of inflammatory bowel disease primarily characterized by ulcerative erosion [1, 2]. The course of UC is long and often recurs [3, 4]. The worldwide prevalence of UC ranges from 5.50 to 24.30/10000, whereas the prevalence of UC in mainland China is about 11.60/10000, which may be underestimated [5].

*Quercus infectoria* Olivier is a small tree or a shrub present in Greece, Asia Minor, Syria, and Iran. The tree grows galls due to infections by the *Cynips gallaetinctoriae* wasp [6]. *Q*. *infectoria* galls (QIGs, also known as Galla Turcica) have remarkable medicinal value and have been pharmacologically deciphered to have astringent, antidiabetic, antitremorine, local anesthetic, antipyretic, and anti-Parkinsonian properties [7]. The main constituents of QIG are tannins (50%–70%) and small amounts of free gallic acid and starch. QIG has been widely used as folk medicine to treat various illnesses, including swelling, inflammation, teeth infection and oral cavity, acute diarrhea, and bleeding [8]. QIG also has a long history in the treatment of UC. The aqueous extract of QIG has an anti-UC effect [9].

At present, QIG for UC treatment is administered as follows: direct swallowing of water decoction, enema, and local application of shredded crude drugs into suppositories. However, after suppository melting, small diffusion area, irregular absorption, and poor patient compliance make coming into contact with UC dispersed lesions extensively challenging [10]. Although the enema fluid is widely distributed, QIG easily leaks, and the action time and dosage of the drug cannot be well controlled [11]. Thus, QIG is inconvenient to use.

The rectal thermosensitive in situ gel has corresponding conditions at average ambient temperature and body temperature. Under the solution-gel transition characteristics, rapid gelation can be achieved at the administration location to achieve location adhesion, effectively prevent drug leakage, prolong retention time, improve the convenience of drug use, and realize location and continuous administration [12]. The thermosensitive in situ gel is expected to resolve the shortcomings of existing rectal dosage forms, effectively reduce the excretion of drugs after an enema, and form a drug reservoir on the surface of ulcers. The unique structure of the gel endows the drug with sustained-release performance to prolong the action time of the drug, improve the curative effect, reduce the discomfort of patients, and increase compliance.

The most popular base of thermosensitive in situ gel is poloxamers (triblock copolymers of poly(oxyethylene)-poly(oxypropylene)-poly(oxyethylene) [PEO-PPO-PEO]). Poloxamers comprise a central block of hydrophobic polypropylene oxide (PPO) surrounded on both sides by the blocks of hydrophilic polyethylene oxide (PEO) [13]. Poloxamers have good water solubility, surface activity, and reverse thermosensitive gelling properties and are safe. At a suitable concentration, the poloxamer system forms micelles in water with a dehydrated PPO chain as core and a hydrated PEO chain as shell [14]. With increasing temperature, the entanglement and stacking between micelles intensify, and the system changes from solution to solidification [15]. The gelling temperature (*T*_sol-gel_) of the system is affected by the PEO/PPO ratio. Therefore, *T*_sol-gel_ of the system can be changed by adjusting the ratio of the poloxamer homolog [16]. Some mucoadhesive polymers, such as sodium alginate, hydroxypropyl methyl cellulose (HPMC), and carboxymethylcellulose sodium (CMC-Na), are commonly needed to increase the adhesion of the drug in the intestine [17].

Therefore, in this experiment, the aqueous extract of QIG is used as primary drug to develop the thermosensitive in situ gel of QIG, improve the existing dosage forms of QIG, enrich the range of dosage forms combined with the reality of patients, flexibly choose the range of dosage forms, and provide a basis for expanding the scope of personalized treatment.

## 2. Materials and Methods

### 2.1. Materials

QIGs were purchased from Guangzhou and identified at the Xinjiang Medical University with voucher number 20190809. Poloxamer 407 (P407; Solarbio, China), poloxamer 188 (P188; Solarbio, China), CMC-Na (Solarbio, China), gallic acid (assigned purity >98%, Solarbio, China), HPMC (Macklin, China), methanol (GR, Sigma-Aldrich, USA), phosphoric acid (AR, Tianjin Zhiyuan Chemical Reagent, China), phosphate-buffered saline (PBS) powder (pH 7.3; ORIGENE, China), and new indocyanine green (IR820) (Macklin, China) were used in experiments.

### 2.2. Preparation of QIG Extract

Aqueous extraction was performed using a modified method reported in the literature [18]. QIG was pulverized into powder. About 200 g QIG passed into an 80-mesh sieve was collected and added with eightfold amount of distilled water. The mixture was soaked for 1 h and subjected to reheating reflux (Great Wall, China) three times for 0.5 h each time. The extract was filtered using Whatman filter papers no. 4 (Cytiva, China, 20–25 *μ*m), and filtrates were concentrated using a rotary evaporator (EYELA, China) at 55°C. The concentrated solution was collected in an evaporation plate, evaporated in the water bath (Jiangsu Jinyi Instrument, China) maintained at 80°C, dried, mashed, placed in a brown glass bottle, and stored in a refrigerator maintained at 4°C.

### 2.3. Preparation of QIG Thermosensitive In Situ Gels

The gel was prepared by the cold method after modification [19]. First, P188, P407, and HPMC were slowly added to the aqueous extract of QIG at a slow speed with magnetic stirrer (Tianjin Medical Instrument Factory, China) and continuously stirred for 5 min in a cold-water bath. Then, mixtures were refrigerated at 4°C for 24 h for full swelling and to obtain a clear and well-dispersed QIG loaded gel solution.

### 2.4. Measurement of *T*_sol-gel_

*T *_sol-gel_, also known as phase transition temperature, is the most important evaluation index of thermosensitive in situ gels. The test tube inversion and the agitator methods are commonly used to determine *T*_sol-gel_. However, when using the agitator method to determine gels, the accuracy of the results is challenging to ensure due to incomplete gel contact, uneven rotor rotation, and heating. Therefore, the test tube inversion method with relatively high accuracy was selected to determine *T*_sol-gel_ in this experiment.

*T *_sol-gel_ of QIG thermosensitive in situ gel was determined by the inverted test tube method [20]. About 2 ml of different gel solutions under cold storage was placed in a glass tube with the same wall thickness and preheated in a water bath maintained at 15°C. The water bath surface was higher than the gel surface. The heating rate was 1°C·min^−1^. The test tube was collected from the water bath every 20 s and tilted slightly to check the fluidity of the gel solution and presence of gelation until the gel was suspended in the test tube after the test tube was inverted. This temperature was *T*_sol-gel_ of the gel solution.

### 2.5. Screening of Bioadhesive Materials

In the mixed solution, P407 (24% [w/w]), P188 (1% [w/w]), HPMC, and CMC-Na were added to prepare solutions with concentration of 1%, and *T*_sol-gel_ was measured by the method described in Section 2.4.

### 2.6. Single-Factor Test

First, P407 blank and drug-loaded gel solutions with different mass concentrations (18%–26%) were prepared, and *T*_sol-gel_ was determined. Second, the concentration of P407 was fixed at 24%, and P188 (1%–5%) was added to prepare drug-loaded thermosensitive gel, and the gel temperature was determined. Finally, the concentrations of P407 and P188 were fixed at 24% and 2%, respectively, and the thermosensitive gel was prepared with 0.3%–1.5% HPMC. Then, the gel temperature was determined.

### 2.7. Formulation Optimization Based on BBD-RSM

The orthogonal test and response surface method (RSM) are commonly used in formulation process screening, and the RSM can obtain more accurate factor levels and has better experimental results than the orthogonal method [21]. Therefore, in this experiment, the best formulation process was selected by the Box-Behnken design response surface method (BBD-RSM). BBD is one of the most adequate techniques for analyzing the data and optimizing the formula [22].

#### 2.7.1. Design of Experiments

Based on single-factor test results, the formulation of QIG thermosensitive in situ gel was optimized using the BBD-RSM. In the design phase, the contents of P407 (24%–26% [w/w], X_1_), P188 (1%–4% [w/w], X_2_), and HPMC (0.3%–1.2% [w/w], X_3_) were used as the independent variables. *T*_sol-gel_ was used as the dependent variable, with 3-factor and 3-level star point design experiment to optimize the thermosensitive gel formulation. The coded levels are −1, 0, and +1, and test factor levels and results are shown in Tables 1 and 2.

#### 2.7.2. Analysis of Variance (ANOVA) and Significance Test

Statistical analysis was performed using the Expert-Design® 10.0.1.0 software. The relationship between independent and dependent variables was analyzed by the twice polynomial regression. The correlation coefficient (*R*^2^) and confidence *P* values of the equations were used as criteria for model fitting.

#### 2.7.3. Model Fitting

The data in Table 3 were fitted by quadratic polynomial stepwise regression fitting by using the DesignExpert 10.0.1 software. One of the factors was fixed, and the two-dimensional contour map and three-dimensional response surface map of the two other factors to *T*_sol-gel_ were drawn.

#### 2.7.4. Prediction and Validation of Optimal Formulations

Given that the average human body temperature was 37°C, the gelation temperature was set to 33°C to ensure that the thermosensitive in situ gel was liquid at room temperature and could gel quickly at body temperature. *T*_sol-gel_ of 33°C was used as the target temperature and substituted into the simulation equation. Three representative formulas in the optimized formula were selected, and three copies were prepared. The actual *T*_sol-gel_ was measured, and the accuracy of the optimized method was verified and evaluated.

### 2.8. *In Vitro* Drug Release

The *in vitro* release test was carried out in the TP-6 Franz diffusion cell [23] (XZH, China). The semipermeable membrane was clamped between the supply and receiving cells, and 17 ml PBS with pH 7.3 was injected into the receiving cell. After placing magnetic stirrer, the receiving pool drained out the air bubbles. About 1 ml formulation 1 gel was precisely added to the supply pool, and the gel was obtained by heating in an oven at 37°C for 2 min and immediately placed in a water bath with temperature of 37°C ± 0.5°C and rotational speed of 200 r·min^−1^. About 600 *μ*l was sampled at 0.5, 1, 2, 4, 6, 8, 10, 12, and 24 h, and then 600 ml 37°C PBS solution was immediately add. The released liquid was filtered by a 0.22 *μ*m microporous membrane. The peak area of the device was measured by HPLC (Agilent1260 Infinity HPLC, Agilent Technology, USA). The cumulative percentage of drug release (%) was calculated, and the release curve was fitted and analyzed. The cumulative release was calculated in accordance with the following equations:

(1) $$\begin{array}{c} Q_{n}=C_{n}\times V_{n}+\sum_{i=1}^{n-1}C_{i}\times V_{i}, \end{array}$$

(2) $$\begin{array}{c} Q=\frac{Q_{n}}{Q_{0}}, \end{array}$$

where *Q*_*n*_ is the cumulative release of the *n*th sampling point, *C*_*n*_ is the concentration of the *n*th sampling point, *V*_*n*_ is the volume of the dissolution medium, *C*_*i*_ is the *i*-th sampling point concentrations, *V*_*i*_ is the *i*-th sampling point sampling volume, and *Q*_*0*_ is the content of gallic acid in the gel.

### 2.9. Analysis of Gallic Acid in the Release Cell

Analysis was performed on an HPLC system. Chromatographic separation was performed on the 1260 Agilent HPLC system consisting of a UV detector set on 251 nm and a C_18_ column (250 mm × 4.6 mm, 5 *μ*m, Waters, USA). The mobile phase consisted of 10% (v/v) methanol and 90% (v/v) phosphoric acid solution (0.2% [v/v]). The mobile phase was delivered at a constant flow rate of 1 ml·min^−1^. Moreover, the injection volume was 10 *μ*l for all samples. Method validation was performed in accordance with the International Conference on Harmonization guidelines [24].

**Figure 1:**
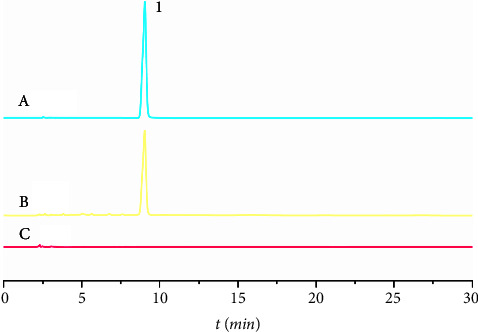
The chromatograms of standard (a), sample (b), and blank (c) solution. 1 is the gallic acid peak.

Specificity, linearity, precision, accuracy, limit of detection (LOD), and limit of quantification (LOQ) were evaluated. A certain concentration of the gallic acid standard solution (A), QIG sample solution (B), and blank sample solution (C) were injected into the HPLC system with the same operation. The chromatogram is shown in Figure 1. The peak of gallic acid was well separated and was not affected by excipients. For linearity studies, an eight-point calibration curve was prepared by diluting the standard working solution (160 *μ*g·ml^−1^) with deionized water, and the range of this calibration curve was from 1.25 *μ*g·ml^−1^ to 160 *μ*g·ml^−1^ (*Y* = 15.089X − 33.649, *r* = 0.9992). Interday precision was realized using the same quality control samples (80 *μ*g·ml^−1^) for six successive days, and the relative standard deviation (RSD) was 1.35%. Intraday precision was realized using the same quality control samples (80 *μ*g·ml^−1^) in one day (RSD = 0.79%). Accuracy was determined by the addition of known amounts of GA standard drugs (at 50%, 100%, and 150% levels) to infusion samples in triplicate (RSD = 2.0%). LOD and LOQ were calculated using slope and *Y*-intercept (0.29 and 0.91 *μ*g·ml^−1^).

### 2.10. Morphological Observation and Characterization of the QIG Thermosensitive In Situ Gel with Scanning Electron Microscope (SEM)

The gel was freeze-dried using freeze dryer (LABCONCO, USA) for 24 h into powder, and the appropriate amount of gel powder was sprayed with gold, 15 kV accelerated voltage, and the microstructure and pore size of the gel surface were observed by SEM [25] (JOEL, Japan).

### 2.11. Fourier Transform Infrared (FTIR) Spectroscopy Characterization on QIG Thermosensitive In Situ Gel

FTIR spectra (Shimadzu, Japan) of formulation and excipients separately were obtained. About 2 mg QIG medicine, QIG water extract, blank thermosensitive gel, and QIG thermosensitive gel were separately placed in an agate mortar and added with 200 mg ground KBr. Mixtures were mixed evenly, placed into an ingot shaper, pressed into tablet form with 15 MPa (9t) for about 1 min, collected, and scanned 10 times. The resolution was 4 cm^−1^, and the scanning range was 4000–500 cm^−1^.

### 2.12. Sol Dynamics

The tilting plate method was conducted as follows. The glass container was kept at a tilt angle of 45° at room temperature (25°C) for 5 min. Then, 40 *µ*l sol samples (blank sol and QIG-loaded sol) were dropped onto a glass plate. The time it took for each drop to slip down at a certain length was recorded. The sol kinetic curve was obtained by drawing the time-displacement curve according to these data [26].

### 2.13. Rheological Properties of QIG Thermosensitive In Situ Gel

The viscoelastic properties of hydrogel networks were studied by placing a piece of thermosensitive gel between two plates through deformation-controlled (or stress-controlled) oscillatory experiment [27]. Taking the appropriate gels, carried out on a rheometer (Malvern, UK), table of shear rates (TSR), shear stress ramp (SSR), and three-step test (TST) was tasted under rotational mode (CP4/40 stainless-steel parallel plates). Then, in the oscillation mode (PU40 stainless-steel parallel plate), the sample was scanned by sequences amplitude sweep (AS), frequency scan (FS), and temperature scan (TS).

#### 2.13.1. TSR of the QIG Thermosensitive In Situ Gel

The TSR test of QIG thermosensitive in situ gel was performed at 4°C, 25°C, and 37°C to determine the fluid type of the sample in rotation mode. The shear rate (*γ*) was set to 0.1–100 s^−1^, and the relationship among *γ*, shear stress (*σ*), and viscosity (*η*) was determined.

#### 2.13.2. SSR of the QIG Thermosensitive In Situ Gel

The SSR test of QIG thermosensitive in situ gel was performed at 4°C, 25°C, and 37°C to determine the gel's retention. The relationship between *σ* and *η* was determined by setting *σ* and time to 200 Pa and 100 s, separately.

#### 2.13.3. TST of the QIG Thermosensitive In Situ Gel

Shear thinning materials may have thixotropy, whereas thixotropic materials are continuously undergoing shear thinning. The QIG thermosensitive in situ gel was tested by TST at 4°C, 25°C, and 37°C to determine whether the samples had thixotropy. The relationship between time and viscosity was observed by setting the shear rate and time as 0.1 s^−1^ and 30 s, 100 s^−1^ and 600 s, and 0.1 s^−1^ and 30 s, respectively.

#### 2.13.4. AS of the QIG Thermosensitive In Situ Gel

The QIG thermosensitive in situ gel was tested by AS at 4°C, 25°C, and 37°C to determine the maximum deformation that the internal three-dimensional reticular structure of the gel could bear when destroyed. Measurement conditions were strain amplitude (*γ*^*∗*^) of 0.1%–100% and frequency (*f*) of 1 Hz. Associations between *γ*^*∗*^ and storage modulus (*G*′), loss modulus (*G*^″^), and phase angle (*δ*) were determined.

#### 2.13.5. FS of the QIG Thermosensitive In Situ Gel

Frequency scanning was designed to describe the time-dependent characteristics of the sample in the range of nondestructive deformation. The temperature was controlled at 4°C, 25°C, and 37°C; the strain was 1%; and the frequency was set to 0.1–10 Hz. The frequency of the QIG thermosensitive in situ gel was measured in oscillation mode, and the relationships between *f* and *G*′, *G*^″^, and *δ* were determined.

#### 2.13.6. TS of the QIG Thermosensitive In Situ Gel

The frequency was set at 1 Hz, and the strain was 1%. The sample was heated at a rate of 5°C·min^−1^, and the temperature range was 5°C–50°C. Changes in *G*′, *G*^″^, complex *η* (*η*^*∗*^), and *δ* with temperature were measured.

### 2.14. *In Vivo* Images of the Thermosensitive Gel

NIR fluorescence imaging images were obtained and processed with the IVIS Spectrum (Perkin Elmer, USA) to know the residence time of gels in the rectum. Ten female KM mice (12 weeks old) purchased from the Animal Center of Xinjiang Medical University (Experimental Animal Production License no. SCXK [New] 2018-0002) were used. Mice were randomly divided into the thermosensitive gel and common enema groups. Before the experiment, mice were fasted for 24 h and drank regular water. First, 100 *μ*l sample with mixed IR820 was removed with a pipette and gently inserted into the rectum of the mouse at depth of about 0.5 cm to push the QIG thermosensitive gel and QIG enema. Mice were anesthetized by the intraperitoneal injection of 10% pentobarbital sodium (0.05 ml·g^−1^) to make mice lie on their back and expose their abdomen. Then, the mice were exposed at 0, 1, 2, 3, 4, 6, 8, and 24 h under the same exposure intensity, and fluorescence signal images were collected.

## 3. Results and Discussion

### 3.1. Screening of Bioadhesive Materials

The effect of HPMC on *T*_sol-gel_ was within the range of human physiological temperature. Thus, HPMC was chosen as adhesive (Table 4).

### 3.2. Single-Factor Test

The single-factor test was carried out to reduce the proportion of each auxiliary material. As shown in Table 3, *T*_sol-gel_ decreased with increasing P407 concentration. *T*_sol-gel_ of P407 increased with the accession of QIG extract compared with the blank gel. When the dosage of P407 of QIG-loaded gel was less than 24%, the solution could not be gelled at 37°C. Thus, the range of optimal dosage of P407 was 24%–26%. By comparison, *T*_sol-gel_ increased with the addition of P188 and HPMC. Considering the matching with the dosage of P407, the dosage of P188 was 1%–4%. However, HPMC increased slightly but had a minor effect. Considering the matching with P407 and P188, the optimization range of HPMC dosage was 0.3%–1.2%.

### 3.3. Design of Experiments

#### 3.3.1. ANOVA and Significance Test

ANOVA was used to analyze experimental data (Table 5). The model's *P* < 0.0001, indicating there is a pretty significant difference. *R*^2^ was 0.9891, and the adjusted *R*^2^ was 0.9750, pointing that this model could illustrate the change of 98.9% response value and reflect the relationship among P407 (X_1_), P188 (X_2_), HPMC (X_3_), and *T*_sol-gel_ (*Y*). The lack-of-fit *P* was 0.8975 > 0.05, which was not significant. Thus, no misfit factor was observed. X_1_, X_2_, and X_3_ had significant effects on *T*_sol-gel_.

#### 3.3.2. Model Fitting

**Figure 2:**
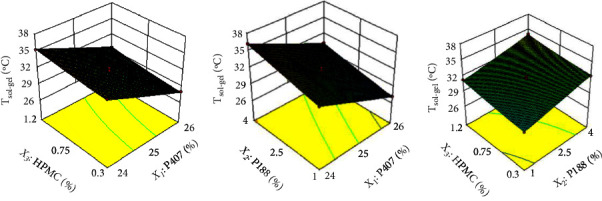
Contour plot and response surface of effect of variables X_1_, X_2_, and X_3_ on *T*_sol-gel_.

The result of the fitting equation was as follows: *T*_sol–gel_ = 31.58 – 3.00X_1_ + 1.48X_2_ + 0.73X_3_ + 0.000_2_ + 0.100_3_ − 0.050_3_ + 0.035X_1_^2^ + 0.085X_2_^2^ − 0.19X_3_^2^. The two-dimensional contour map and the three-dimensional effect map of the experimental results were drawn using the DesignExpert 8.0.6 software (Figure 2).

#### 3.3.3. Prediction and Validation of Optimal Formulations

Table 6 shows that the deviation between the measured and predicted values of the gel temperature of each formulation was less than 2%. This result indicated that the fitting equation had good predictability.

**Figure 3:**
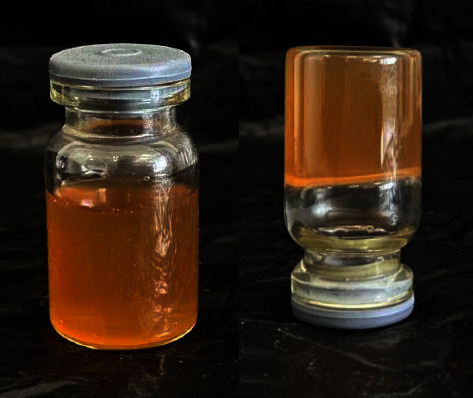
QIG thermosensitive in situ gel morphology.

At room temperature, the QIG thermosensitive in situ gel is a yellow uniformly dispersed solution with good fluidity. The gel is yellow and semisolid above the phase transition temperature (*T*_sol-gel_ ≥ 32.8°C) (Figure 3).

### 3.4. Release Performance

**Figure 4:**
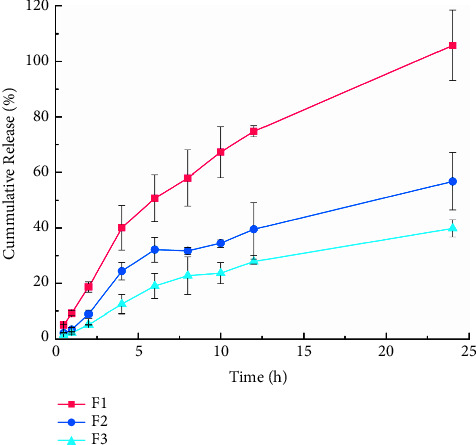
*In vitro* cumulative release and tine curve of gallic acid in QIG thermosensitive gels ($\bar{x}$ ± S, *n* = 3).

As displayed in Figure 4, the release curves of the 3 formulations did not coincide and were fitted by zero-order kinetics, Ritger-Peppas, Higuchi, and first-order kinetic distribution model (OriginPro 8.0 software). The goodness-of-fit was measured by parameters, such as correction determination coefficient (*R*^2^) and analysis of variance (*P*). *R*^2^ is closer to 1, and *P* < 0.05 indicates that the fitting is statistically significant. As shown in Table 7, the first-order kinetic distribution model was the best to fit the release curve of each formulation. *T*_50_ indicated the time taken to release 50% (Table 8) and was more than 3 h, showing apparent sustained-release performance. Given that F1 was entirely released within 24 h and the shared key quality attributes could be achieved using the least number of excipients, F1 was the optimal formulation.

### 3.5. Morphological Observation and Characterization of the QIG Thermosensitive In Situ Gel with SEM

**Figure 5:**
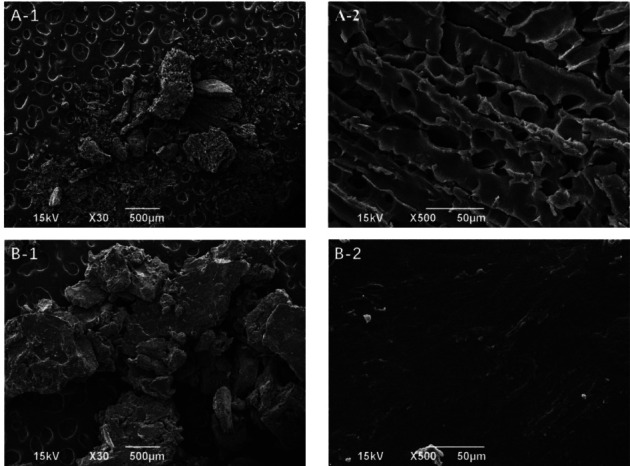
SEM image of blank (a) and loading QIG (b) thermosensitive gel.

As shown in Figure 5, the blank gel (A) had multiple round holes, a loose lamellar structure, void penetration, uniform internal structure, and porous connected mesh. The QIG in the QIG-loaded gel (B) was uniformly dispersed in the lamellar gel structure, and gel pores were filled, indicating that the aqueous QIG extraction could uniformly disperse and fill in the mesh and pores of the gel base surfaces. These physical changes led to the strength of the gel and determine the release law of the QIG.

### 3.6. FTIR Spectroscopy of the QIG Thermosensitive In Situ Gel

**Figure 6:**
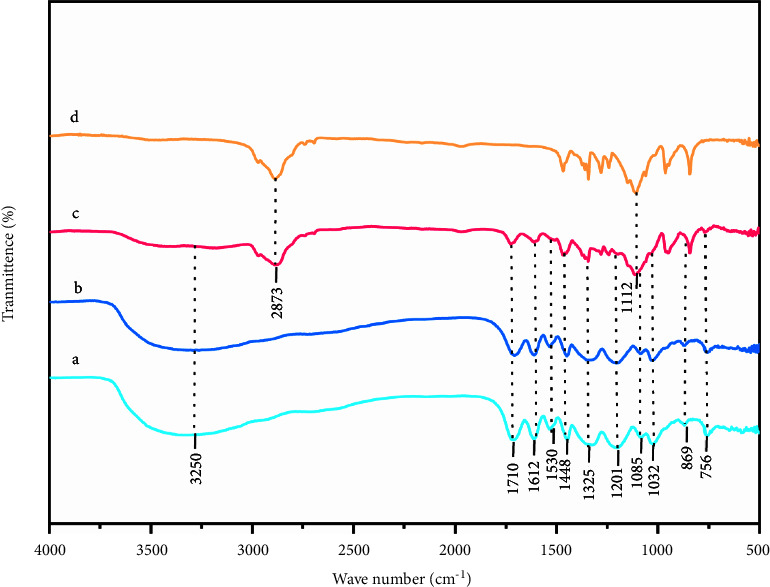
FTIR spectra of QIG herbs (a), QIG aqueous extract (b), QIG-loaded gel (c), and blank gel (d).

The FTIR spectra of the raw medicinal powder, aqueous extract, QIG-loaded gel, and blank gel are shown in Figure 6. The spectrum, which included multiple characteristic absorption peaks, was complex. A series of high-intensity characteristic absorption was found in the 1710 to 1448 cm^−1^ region. The peak at 1708 cm^−1^ was related to the stretching vibration of C=O in the gallic phthalein group [28]. Given the stretching vibration of the C=C group and the deformation and stretching vibrations of the aromatic skeleton and C-H in the plane, a characteristic peak appeared at 1612–1530 and 1448 cm^−1^ [28], and the absorption bands at 1325 and 1201 cm^−1^ were related to the symmetrical in-plane bending vibrations of CH_3_ and CH_2_ [29].

Therefore, excipients did not affect the characteristic functional groups of the API, ensuring the maximum efficacy of the API.

### 3.7. Sol Dynamics

A certain concentration of P407 had reversible temperature-sensitive gelling properties. P407 was liquid at low temperature, became semisolid when the system rose to a certain temperature, and changed back to liquid when the temperature decreased [30].

**Figure 7:**
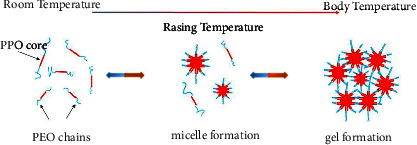
Gelation mechanism of poloxamer.

The main mechanism of gelation in P407 solution was micelle stacking and entanglement. P407 formed a spherical micelle with hydrophobic PPO block as core and wrapped in hydrophilic PEO shell. With increasing temperature, a high polymer concentration resulted in high number of micelles and probability of contact and entanglement. Thus, the gelation temperature was dependent on concentration [31, 32], as shown in Figure 7.

**Figure 8:**
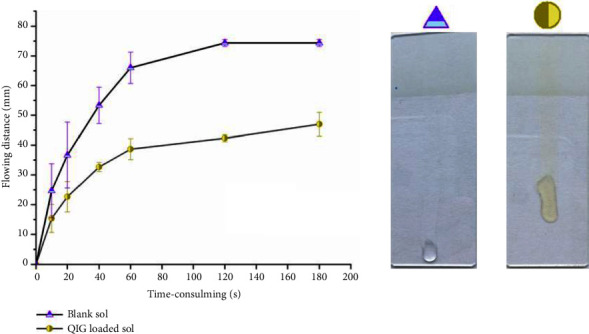
Leakage (mm) of QIG loaded and unloaded gels after 3 min in slide surface.

Based on the above theory, the QIG-loaded gel took more time to complete the same distance of sliding than blank gels. A plausible explanation was that, with the addition of aqueous QIG extract, the water molecules on the PEO blocks became tightly entangled, resulting in difficulty of removing water molecules from PEO blocks. This phenomenon led to a slow kinetic rate of the sol. The addition of the QIG to the sol had a significant effect on the kinetics of the sol. The addition of QIG decreased the kinetic rate of sol, improved the adhesion, and then increased the QIG release rate in the gel (Figure 8).

### 3.8. Rheological Properties of the QIG Thermosensitive In Situ Gel

#### 3.8.1. TSR of the QIG Thermosensitive In Situ Gel

The behavior of the sample in different processes was simulated by *γ*. A very low *γ* (∼0.001 s^−1^) was used to evaluate the stability and quality of the sample, intermediate *γ* (∼10 s^−1^) was used to evaluate pumpability and paintability, and low *γ* (∼1 s^−1^) was used to evaluate the fluidity. High *γ* (∼100 s^−1^) was used to determine whether the sample was too thick to spread. The three general flow behaviors were Newtonian, pseudoplastic, and dilatant plastic fluids.

**Figure 9:**
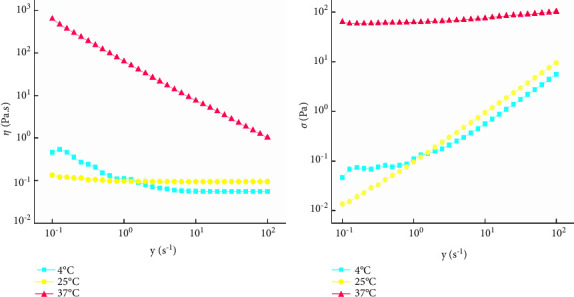
Variation curves of *γ*, *σ*, and *η* of QIG thermosensitive in situ gel at different temperatures.

When the *γ* table was at 4°C and 25°C, *σ* of the QIG thermosensitive in situ gel increased linearly with increasing *γ*, whereas *η* remained unchanged. This result showed that the gel had the properties of a Newtonian fluid. However, at 37°C, *σ* and *γ* of the sample tended to be stable, and sample *η* decreased with increasing *γ*, indicating a pseudoplastic fluid. The QIG thermosensitive in situ gel was inferred to show the state of pseudoplastic fluid after *in vivo* administration. Results are shown in Figure 9.

#### 3.8.2. SSR of the QIG Thermosensitive In Situ Gel

**Figure 10:**
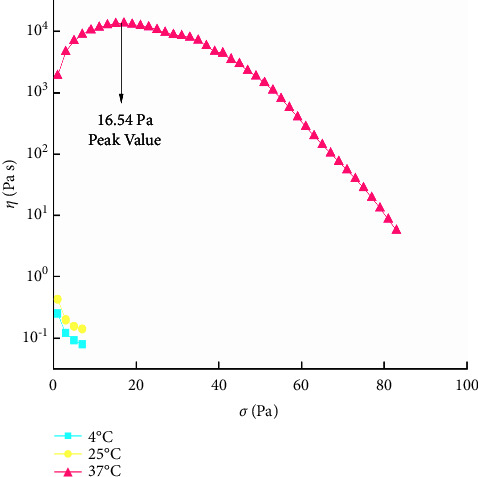
Variation curves of *σ* and *η* of QIG thermosensitive in situ gel at different temperatures.

The QIG thermosensitive in situ gel had no peak value at 4°C and 25°C, indicating no yield stress. This finding was consistent with the results of TSR, and the sample cannot flow. At 37°C, the curve increased first and then decreased, and the peak value was evident.  Figure 10 shows that the gel structure of the sample was formed and that the gel needed particular stress to flow. A special retention force in the drug administration site was preliminarily predicted.

#### 3.8.3. TST of the QIG Thermosensitive In Situ Gel

**Figure 11:**
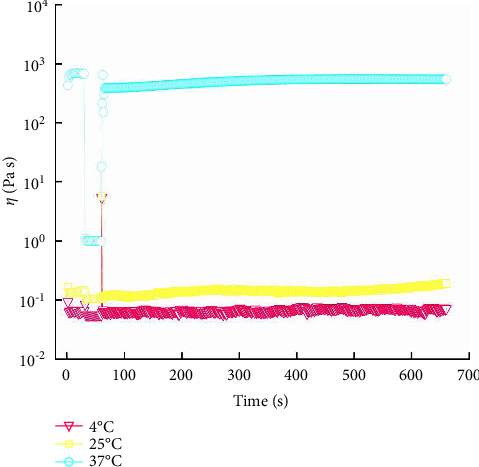
Variation curve of stepped shear rate *γ* and *η* with time at different temperatures of QIG thermosensitive in situ gel.

Figure 11 shows the change in *η* with *γ* tending to be a straight line, indicating that the sample is Newtonian fluid at 4°C and 25°C. However, the sample has a small shear thinning process at 37°C, it can recover instantly after the high shear process, and the relationship between time and viscosity tends to be linear. Therefore, the gel has no thixotropy and is not time-dependent.

#### 3.8.4. AS of the QIG Thermosensitive In Situ Gel

**Figure 12:**
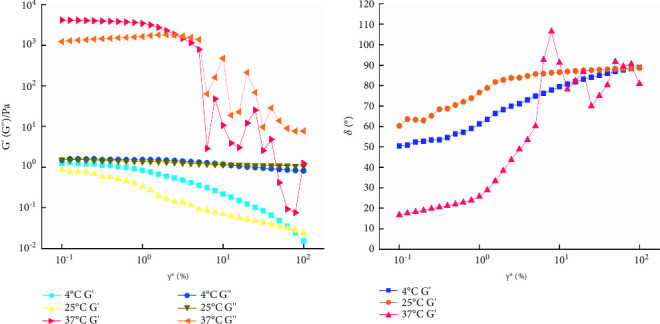
The curves of *γ*^*∗*^ and *G*′, *G*″, and *δ* of galactic temperature-sensitive in situ gels at different temperatures.

Results are shown in Figure 12. At 4°C and 25°C, *G*^″^ > *G*′, and *δ* > 45° indicated that the lost part of the sample was larger than the stored part having fluid characteristics, which is a viscoelastic liquid, and also *G*′*G*^″^ does not change with the increase of *γ*^*∗*^. However, at 37°C, *γ*^*∗*^ < 1%, *G*′ > *G*^″^, and *δ* < 45° indicated that the elasticity of the sample was dominant, representing a gel structure. When *G*′ = *G*^″^ the gel structure is destroyed, *G*′ < *G*^″^ and *δ* > 45° indicated the viscosity of the sample was dominant, and it shows up fluid properties. Therefore, all dynamic oscillation experiments should control *γ*^*∗*^ within 1%.

#### 3.8.5. FS of the QIG Thermosensitive In Situ Gel

**Figure 13:**
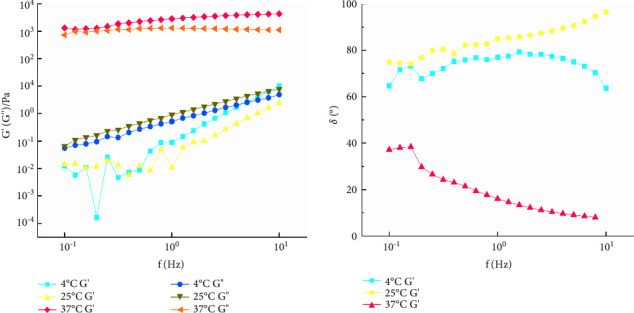
The curves of *f* and *G*′, *G*″, and *δ* of galactic temperature-sensitive in situ gels at different temperatures.

At 4°C and 25°C, *G*^″^ was always larger than *G*′, and *δ* was much larger than 45°, showing evident viscous fluid characteristics. At 37°C, samples *G*′ and *G*^″^ evidently increased by about four orders of magnitude. *G*′ > *G*^″^ and *δ* < 45° showed evident elastic properties, and no significant frequency dependence was observed, indicating that the sample always had a stable crosslinking structure (Figure 13).

#### 3.8.6. TS of the QIG Thermosensitive In Situ Gel

**Figure 14:**
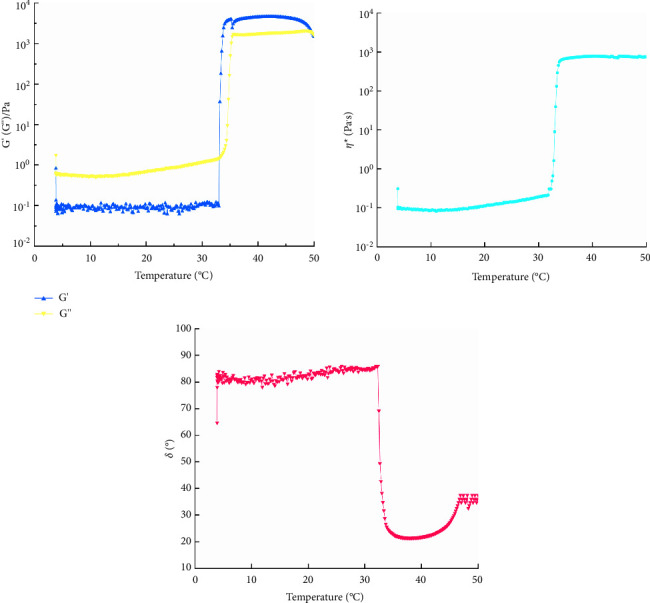
The curves of *f* and *G*′, *G*″, and *δ* of galactic temperature-sensitive in situ gels at programmed heating.

Figure 14 shows that, with increasing temperature, *G*^″^ and *η*^*∗*^ increased, whereas *δ* decreased. At about 32°C, *G*′ and *G*^″^ intersected, and the intersection point was its phase transition temperature (*T*_sol-gel_). Before *T*_sol-gel_, sample *G*^″^ > *G*′, *η*^*∗*^ was small, and *δ* > 45°. These results indicated that the sample had *G*^″^ and low *η*, showing prominent fluid properties. It gradually increased *G*′ > *G*^″^, *η*^*∗*^ increased sharply, and *δ* < 45° after *T*_sol-gel_. This result indicated that the gel structure was formed, and the gel structure *G*^″^, *η*^*∗*^, and *δ* tended to be flat, which proved that the viscoelasticity of the gel tended to be stable.

### 3.9. *In Vivo* Images of the Thermosensitive Gel

**Figure 15:**
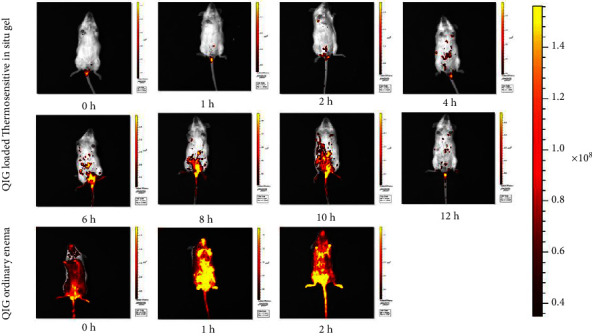
NIR fluorescence of KM mice administered with IR820-loaded QIG thermosensitive in situ gel and IR820-loaded ordinary enema at different time points.

The duration of the material after enema *in vivo* should be determined for controlled drug release. The distribution and retention of QIG in mice in the ordinary and thermosensitive gel enema groups could be observed through fluorescence imaging. In Figure 15, partial leakage could be seen with naked eyes after administration in the ordinary enema group. Fluorescence signals could be observed on the back paw, fur, and tail of mice. In the thermosensitive gel enema group, QIG extended and gelled from rectum to colon, and the fluorescence signal could still be observed at 12 h. In the ordinary enema group, QIG was almost distributed all over the body at 1 h. This result indicated that the temperature-sensitive gel could prolong the local retention time of new indocyanine green.

## 4. Conclusions

The QIG thermosensitive in situ gel was prepared by the cold method. First, the preparation process used *T*_sol-gel_ as index. The number of excipients was investigated and selected by the single-factor test. Second, on the basis of the single-factor experiment, the dosages of P407, P188, and HPMC were optimized by BBD-RSM. In accordance with the optimized screening results of the number of excipients and by using the drug release performance *in vitro* as index, the optimal preparation process of QIG thermosensitive in situ gel was determined. The best preparation results were as follows: P407 (24.07% [w/w]), P188 (1.22% [w/w]), and P188 (0.60% [w/w]), and *T*_sol-gel_ was 32.8°C ± 0.4°C. The *in vitro* cumulative release curve of QIG thermosensitive gel followed the first-order kinetics equation: *Q* = 119.68 × (1 − e^−0.09t^) (*r* = 0.9968) and it was completely released within 24 h. SEM results indicated that the blank gel had a porous connected three-dimensional network structure. QIG was uniformly dispersed in the gel lamellar structure, and gel pores were filled. This change might increase the strength of the gel. FTIR analysis verified that excipients had no effect on the characteristic peak of QIG, and rheological studies confirmed that the QIG thermosensitive in situ gel had Newtonian fluid properties, no yield value and thixotropy at 4°C and 25°C, pseudoplastic fluid property, and yield value without thixotropy at 37°C. *In vivo* imaging showed that the fluorescence signal could still be observed in the QIG temperature-sensitive in situ gel group at 12 h and that IR820 was almost all over the body at 1 h in the ordinary enema group, confirming that the QIG thermosensitive in situ gel could prolong the local retention time of IR820.

In summary, the prepared QIG thermosensitive in situ gel has the functions of temperature sensitivity, sustained release, strong spreadability, and high retention. In addition, the stability, quality standard, rectal mucosal irritation, pharmacodynamics, and pharmacokinetics of the QIG thermosensitive in situ experimental gel test should be further studied to improve the preclinical research.

## Figures and Tables

**Table 1:** Independent variables and experimental level in this study.

Independent variable	Factors	Experimental level		
		Low (−1)	Middle (0)	High (+1)
P407/%	*X*1	24	25	26
P188/%	*X*2	1	2.5	4
HPMC/%	*X*3	0.3	0.75	1.2

**Table 2:** Results of Box-Behnken design response surface methodology ($\bar{x}$ ± S, *n* = 3).

Run	*X*1 (%)	*X*2 (%)	*X*3 (%)	Experimental *T*_sol-gel_ (°C)
1	+1	0	+1	29.5 ± 0.1
2	0	0	0	31.4 ± 0.5
3	0	0	0	31.7 ± 0.2
4	0	+1	−1	32.5 ± 0.3
5	−1	−1	0	33.2 ± 0.4
6	0	0	0	31.7 ± 0.4
7	−1	0	+1	35.3 ± 0.5
8	+1	+1	0	30.2 ± 0.2
9	−1	+1	0	36.2 ± 0.4
10	0	+1	+1	34.1 ± 0.3
11	−1	0	−1	34.3 ± 0.2
12	+1	−1	0	27.2 ± 0.4
13	0	−1	−1	29.5 ± 0.2
14	0	−1	+1	31.3 ± 0.4
15	0	0	0	32.2 ± 0.1
16	0	0	0	30.9 ± 0.3
17	+1	0	−1	28.1 ± 0.2

**Table 3:** Results of single-factor investigation ($\bar{x}$ ± S, *n* = 3).

Inspection project						
P407			P188		HPMC	
Concentration/%	*T*_sol-gel_ (QIG loading)/°C	*T*_sol-gel_ (blank)/°C	Concentration/%	*T*_sol-gel_ (QIG loading)/°C	Concentration/%	*T*_sol-gel_ (QIG loading)/°C
18	>37	>37	1	31.9 ± 0.2	0.3	33.5 ± 0.4
19	>37	36.2 ± 0.2	2	33.5 ± 0.3	0.6	34.1 ± 0.2
20	>37	33.1 ± 0.2	3	33.9 ± 0.2	0.9	34.9 ± 0.2
21	>37	30.1 ± 0.5	4	35.4 ± 0.2	1.2	35.4 ± 0.4
22	>37	27.4 ± 0.3	5	36.8 ± 0.1	1.5	36.2 ± 0.2
23	>37	25.3 ± 0.3	6	>37		
24	31.1 ± 0.3	22.7 ± 0.2				
25	28.4 ± 0.4	19.5 ± 0.4				
26	25.2 ± 0.2	15.7 ± 0.2				

**Table 4:** Effects of different bioadhesives on exterior, pH, and *T*_sol-gel_ ($\bar{x}$ ± S, *n* = 3).

Bioadhesives	Evaluation index		
	Exterior	pH	*T*_sol-gel_/°C
HPMC	Orange, transparent	4.40 ± 0.1	34.40 ± 0.2
CMC-Na	Orange, transparent	4.63 ± 0.2	—

(—) Indicates that 40°C is incoagulated.

**Table 5:** Factor regression coefficients and ANOVA.

Source	Sum of squares	DF	Mean Square	*F*-value	*P* value	Significance
Model	93.85	9	10.43	70.32	<0.0001	Significant
X_1_	72	1	72	485.55	<0.0001	significant
X_2_	17.41	1	17.41	117.37	<0.0001	significant
X_3_	4.2	1	4.2	28.36	0.0011	significant
X_1_X_2_	0	1	0	0	1	
X_1_X_3_	0.04	1	0.04	0.27	0.6195	
X_2_X_3_	0.01	1	0.01	0.067	0.8026	
X_1_^2^	0.0052	1	0.0052	0.035	0.8573	
X_2_^2^	0.03	1	0.03	0.21	0.6643	
X_3_^2^	0.14	1	0.14	0.97	0.3571	
Residual	1.04	7	0.15			
Lack of fit	0.13	3	0.043	0.19	0.8975	Not significant
Pure error	0.91	4	0.23			
Cor total	94.89	16				

**Table 6:** Formulation verification and optimization results ($\bar{x}$ ± S, *n* = 3).

Formulations	P407/%	P188/%	HPMC/%	Observed *T*_sol-gel_/°C	Predicted *T*_sol-gel_/°C	Deviation
F1	24.07	1.22	0.6	33	32.8 ± 0.4	0.6
F2	25.23	3.77	1.17	33	33.6 ± 0.2	1.8
F3	24.96	3.94	0.6	33	33.3 ± 0.5	0.9

**Table 7:** Results of fitting equation.

Formulations	Zero-order kinetic equation		Ritger-Peppas equation		Higuchi equation		First-order kinetic equation	
	*R* ^2^	*P*	*R* ^2^	*P*	*R* ^2^	*P*	*R* ^2^	*P*
1	0.87979	1.1×10^−5^	0.96283	0	0.98563	1.1 × 10^−7^	0.99364	6.5 × 10^−10^
2	0.82669	4.2×10^−4^	0.96509	6.2 × 10^−6^	0.95788	4.5 × 10^−7^	0.96901	1.7 × 10^−7^
3	0.80642	6.2×10^−4^	0.9577	1.6×10^−5^	0.95289	8.9 × 10^−7^	0.98085	3.9 × 10^−7^

**Table 8:** Fitting results of first-order kinetic equation.

Formulations	Fitting equation	*R* ^*2*^	*P*	*T*_50_/h
1	M_t_ = 119.68 (1 − *e*^−0.09t^)	0.99364	6.5 × 10^−10^	3.4
2	M_t_ = 60.05 (1 − *e*^−0.10t^)	0.96901	1.7 × 10^−7^	17.7
3	M_t_ = 37.46 (1 − *e*^−0.10t^)	0.98085	3.9 × 10^−7^	11.1

## Data Availability

The data used to support the findings of this study are available from the corresponding author upon request.
